# Idiopathic Megaduodenum in a Teenager: A Case Report

**DOI:** 10.7759/cureus.51930

**Published:** 2024-01-09

**Authors:** Anthony N Eze, Akachukwu N Eze, Chinecherem M Chime, Fengming Chen, Dimitrios Moris, Robin Schmitz, Tamara N Fitzgerald

**Affiliations:** 1 Department of Surgery, Duke University Medical Center, Durham, USA; 2 Duke Global Health Institute, Duke University, Durham, USA; 3 Department of Medicine, Howard University College of Medicine, Washington, D.C., USA; 4 Department of Pathology, Duke University Medical Center, Durham, USA; 5 Department of Surgery, University of Pittsburgh Medical Center, Pittsburgh, USA

**Keywords:** malnutrition, small bowel obstruction, idiopathic, superior mesenteric artery syndrome, duodenal volvulus, megaduodenum

## Abstract

Megaduodenum is a rare clinical syndrome characterized by significant duodenal dilation, elongation, and hypertrophy. Given its rarity and nonspecific clinical manifestations, megaduodenum may be misdiagnosed, leading to delays in surgical care and increased morbidity. We describe a case of idiopathic megaduodenum in a teenage Caucasian female, who presented with a five-year history of halitosis, recurrent belching, bloating, nausea and vomiting, and postprandial epigastric abdominal pain. She was diagnosed with megaduodenum by dramatic findings on contrast radiography. She developed a duodenal volvulus necessitating emergency exploratory laparotomy, during which a duodenal plication and a side-to-side duodenojejunostomy were performed. Exploratory laparotomy and histopathological analysis were unrevealing of any definitive abnormalities to explain her megaduodenum. Postoperatively, she developed two early small bowel obstructions, both from subsequent adhesions requiring repeat laparotomy with adhesiolysis. She has subsequently recovered without incident. Diagnosis and accurate classification of megaduodenum requires surgical exploration with a full-thickness biopsy and subsequent histopathologic analysis to rule out obstructive or functional disorders of the duodenum. Treatment of megaduodenum depends on the underlying cause and degree of duodenal distention. It is crucial that clinicians are knowledgeable of the various surgical options, their indications, and the potential postoperative complications that may arise.

## Introduction

Megaduodenum is a rare clinical syndrome characterized by significant duodenal dilation (>5 cm diameter), elongation, and hypertrophy [1-3]. Usually, the dilation is limited to the descending, horizontal, and ascending parts (i.e., 2nd-4th) of the duodenum [1-3]. Megaduodenum was first described in 1863 by Rokitansky, but Melchoir authored the first clinical report in 1924 [1,3]. In the last 100 years, only about 60 cases have been described [4]. Megaduodenum has been reported in both adults and children and has no clear gender predominance [1,4,5]. It is especially rare in early childhood but can have a significant and longstanding impact on a child’s growth, development, and overall well-being [1,3-5]. The mean age at diagnosis of patients with megaduodenum is reported as 38 years [5].

Given its rarity and nonspecific clinical manifestations, megaduodenum may be misdiagnosed, as it can mimic other intestinal disorders [4]. Delay in surgical care leads to increased morbidity. Specifically, idiopathic megaduodenum is even more rare and a diagnosis of exclusion. Only 11 cases of idiopathic megaduodenum have been reported in the literature. The first cases of idiopathic megaduodenum were reported in 1985 by Eaves et al., while the most recent report in the literature was by Horvat et al. in 2019. All reported cases of idiopathic megaduodenum were managed via either a duodenal-jejunal bypass alone or a tapering duodenoplasty with a duodenal-jejunal bypass. To facilitate accurate and timely diagnosis and effective management of this rare condition, we describe a case of idiopathic megaduodenum in a teenage girl managed with a duodenal plication and duodenojejunostomy.

## Case presentation

An 18-year-old Caucasian female presented to our clinic with a five-year history of halitosis, recurrent belching, bloating, recurrent nausea and vomiting, back pain, and postprandial epigastric abdominal pain, which improved upon lying down. She denied a history of diarrhea, pancreatitis, or early satiety. While she denied a history of weight loss, she was malnourished with a body mass index of 19 kg/m^2^ (fourth percentile) and labs demonstrated a hypoalbuminemia (1.7 g/dL) and vitamin D deficiency (25.0 ng/mL). All other labs were unremarkable, and she tested negative for celiac disease. She was born at full term with no postnatal issues, and even now as a teenager, she had no significant past medical history and no allergies. She had no history of prior surgery and had no significant family history.

**Figure 1:**
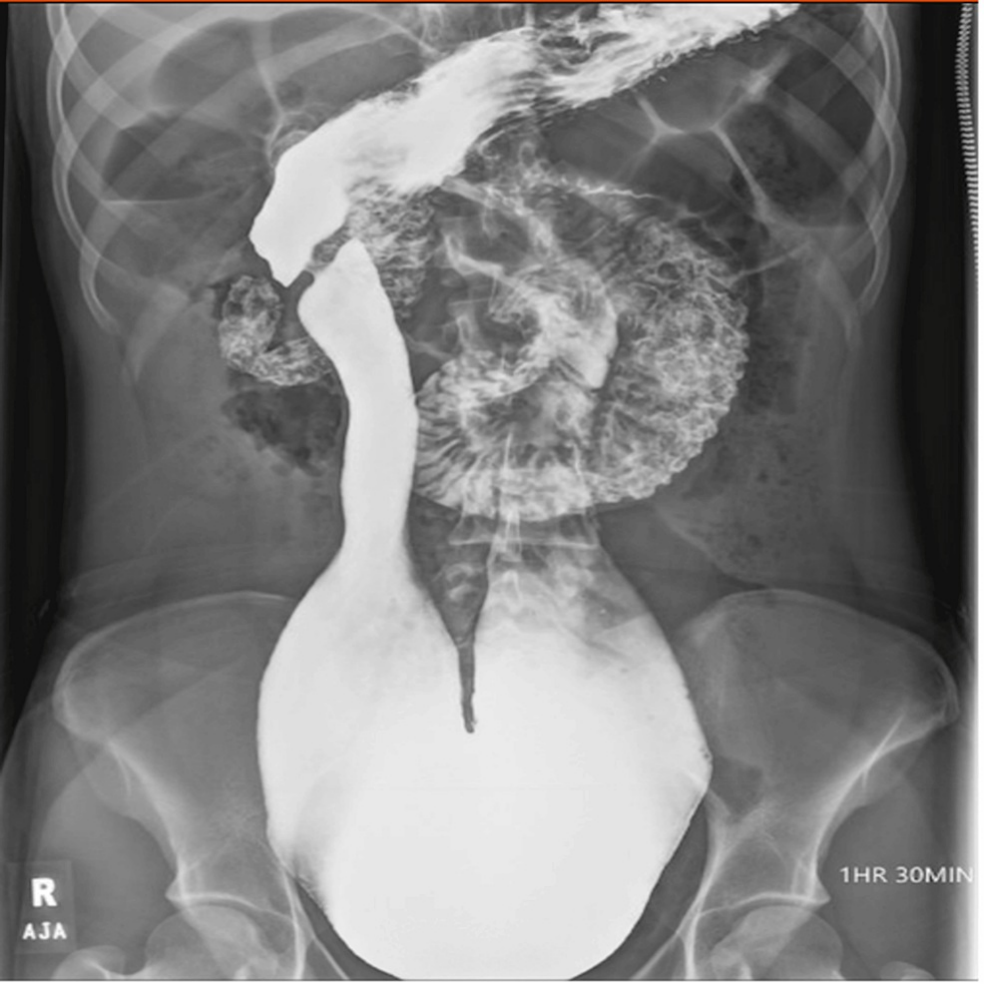
Preoperative small bowel series demonstrating megaduodenum Dilated and elongated duodenum extending into the pelvis. The duodenum measures up to 11 cm along the third portion.

She was referred to our hospital after receiving a grossly abnormal small bowel radiograph series, demonstrating that her duodenum descended into the pelvis and measured up to 11 cm at the third portion (Figure 1).

**Figure 2:**
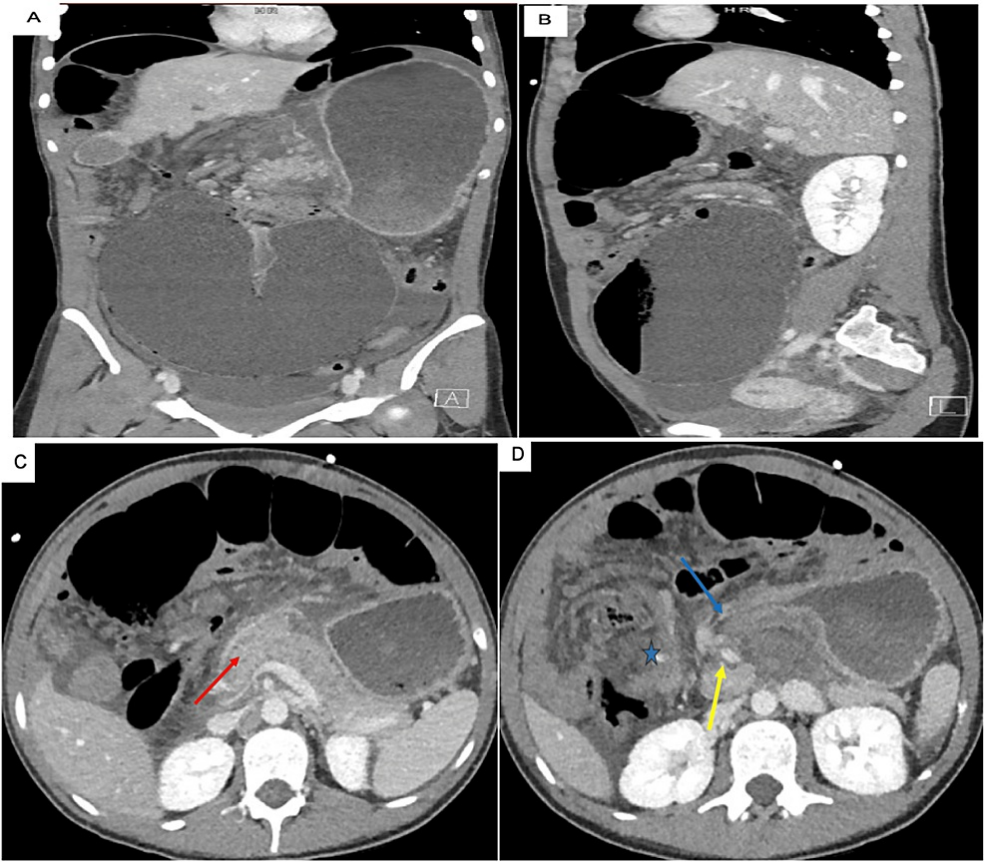
(A) Coronal and (B) sagittal views demonstrating megaduodenum. (C) and (D) Axial views demonstrating duodenal volvulus. A) Coronal and B) sagittal computed tomography images demonstrating megaduodenum extending into the pelvis and closed-loop obstruction from the duodenal volvulus with pneumatosis intestinalis concerning for ischemia. C) Axial view of the duodenal volvulus with a significant mesenteric swirling with the pancreas (red arrow) being pulled into the volvulus. D) Axial view showing the volvulus with the distal stomach (blue arrow) and superior mesenteric artery (SMA) (yellow arrow) being pulled to the right of the midline by the volvulus. The blue star indicates a dilated duodenum with surrounding mesenteric swirling.

Upon our evaluation, her abdomen was distended but nontender. Her case was discussed in a multidisciplinary conference among several surgeons, radiologists, and gastroenterologists, and it was agreed that her duodenum was non-functional and required resection. She was offered immediate surgery, but the family chose to postpone surgery for several weeks given that she was in the middle of her school semester. However, before reaching the date of elective surgery, she emergently presented with severe abdominal pain, nausea, vomiting, and failure to pass gas or stool. Laboratory studies demonstrated electrolyte abnormalities with leukocytosis (14.6/L), elevated amylase (1053 U/L), and elevated lipase (>3500 U/L). All other labs were unremarkable, including total bilirubin, triglyceride, and liver enzymes. She was subsequently found on computed tomography (CT) to have a large duodenal volvulus with pneumatosis intestinalis of the massively dilated duodenum concerning for ischemia (Figure 2). There was significant mesenteric swirling with a portion of the pancreas being pulled into the volvulus, and both superior mesenteric artery (SMA) and the distal stomach were being pulled to the right of the midline.

**Figure 3:**
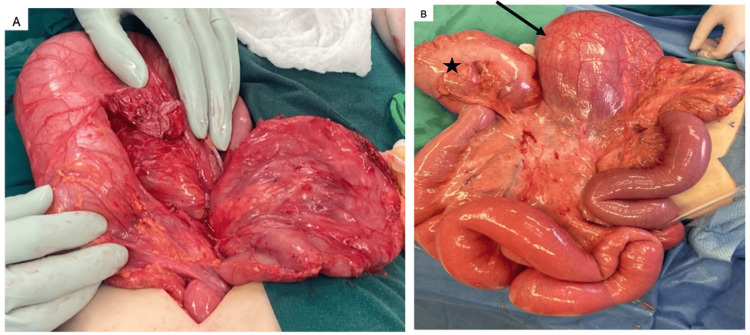
Intraoperative photos A) Intraoperative photo showing the dilated duodenum being pulled out of the pelvis. B) Intraoperative photo demonstrating the megaduodenum after detorsion of volvulus (black arrow). The cecum (black star) has been displaced by the megaduodenum and is not fixed to the retroperitoneum. All bowel is healthy-appearing.

Given these findings, a nasogastric tube was placed, and she was taken for emergency exploratory laparotomy. The procedure began by entering the abdomen via a midline incision. There was minimal torsion of the duodenum, but the duodenum was large and extended down into the pelvis. The duodenum was easily detorsed (Figure 3). The cecum appeared dilated and not fixed in the right lower quadrant, possibly because it had been partially obstructed and displaced by the large duodenum. The remainder of the bowel was loosely fixed by a thin mesentery, but otherwise appeared healthy. The duodenum was kocherized allowing for inspection of the pancreas and the first to third portions and most of the fourth portion of the duodenum. The pancreas appeared healthy. The antimesenteric edge of the distal duodenum near the ligament of Treitz was opened. The lumen was palpated proximally and distally to the jejunum.

**Figure 4:**
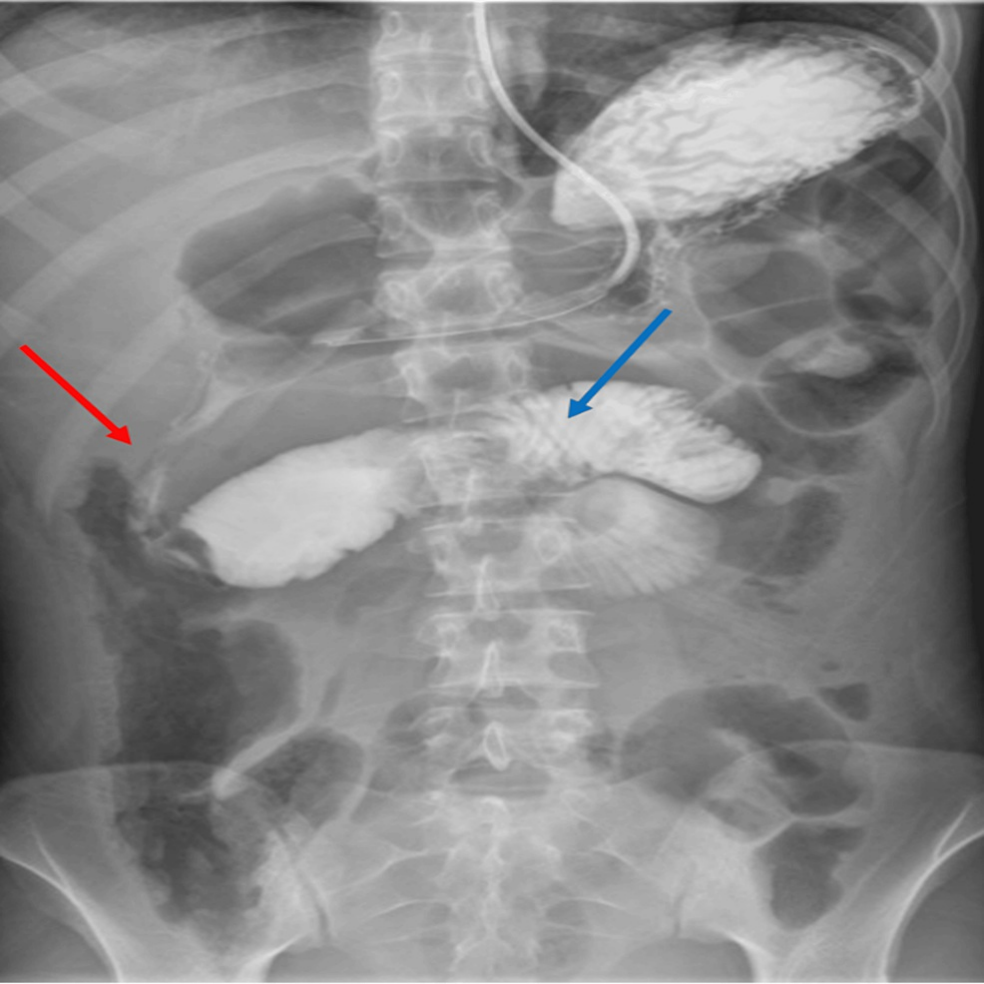
Postoperative upper gastrointestinal series demonstrating new duodenal anatomy with a reduced duodenal caliber after duodenal plication and duodenojejunostomy. The red arrow points to the reduced duodenal caliber. The blue arrow points to the site of the duodenojejunal anastomosis. There was no leak noted.

While no stricture, web, or mass could be palpated, the SMA felt heavy against the duodenum, raising the question for SMA syndrome as a potential cause of her megaduodenum. Plication of the duodenum was performed as follows: a large red rubber catheter was placed into the lumen of the duodenum, and multiple staple loads were used to resect the antimesenteric redundant portion of the duodenum from the pylorus to the duodenal enterotomy by the ligament of Treitz making sure to avoid injury to the biliary drainage system. This significantly reduced the duodenum’s caliber. A duodenojejunal side-to-side anastomosis was performed, bypassing the section of duodenum under the SMA. The abdomen was closed. Given the level of chronic malnourishment, a right internal jugular central venous catheter was placed for postoperative delivery of total parenteral nutrition. A postoperative upper gastrointestinal contrast radiograph series obtained days later demonstrated her new gastrointestinal anatomy and confirmed the absence of any leak (Figure 4). 

**Figure 5:**
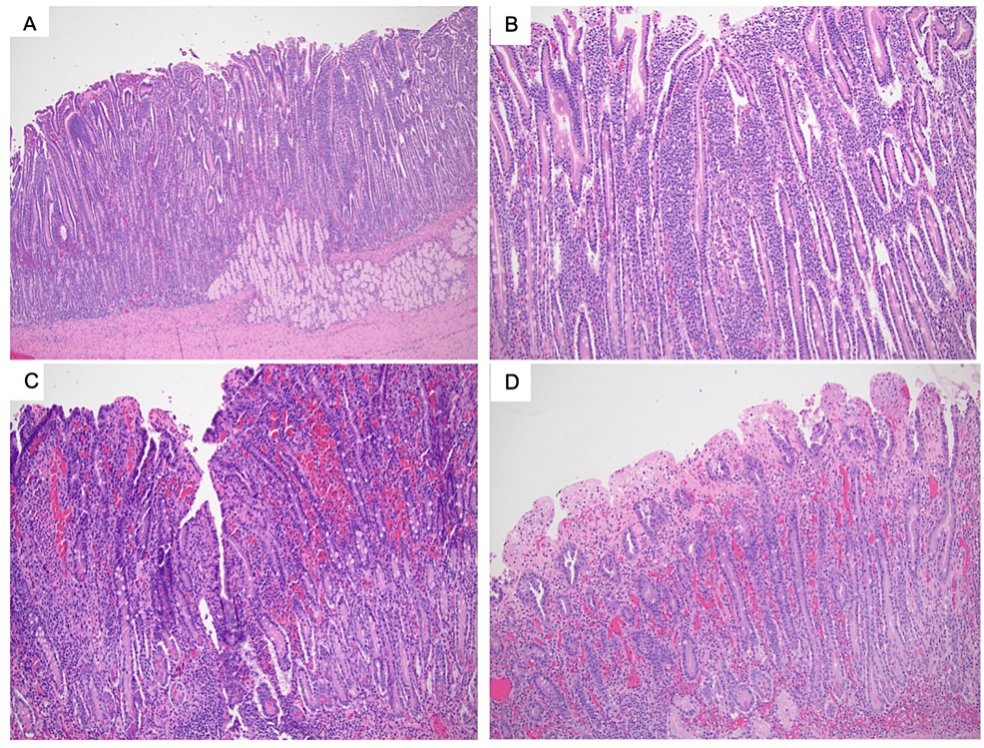
Histopathologic analysis A) Hematoxylin and eosin-stained tissue sections showing the segment of duodenum with villous attenuation and crypt hyperplasia (40x). Also shown are B) mild lamina propria expansion by chronic inflammation (100x), C) focal acute inflammation (100x), and D) focal ischemic changes (100x).

On gross examination, the resected duodenum measured 18.5 cm in length with a 5.7 cm circumference. There was minimal perimesenteric adipose tissue. No masses or nodules were noted. Microscopically, it showed mild lamina propria expansion by acute and chronic inflammation, focal ischemic changes, crypt hyperplasia, and villous attenuation (Figure 5). Although the histopathologic finding is not very specific, it could be seen in patients with malabsorption due to the abnormal small intestinal motility. Postoperatively, she completed four days of total parenteral nutrition supplementation, had return of bowel function, and was subsequently transitioned to a regular diet. She recovered well and was safely discharged home.

Two months later, she was readmitted to our hospital with abdominal pain and distension and was found to have a small bowel obstruction with mesenteric edema. For this, she underwent an exploratory laparotomy with adhesiolysis of a single adhesive band causing an internal hernia. Her bowel was healthy-appearing, but an appendectomy was performed to avoid future diagnostic uncertainties, given the mobility of the cecum. The appendix had no significant pathological findings. Postoperatively, she recovered well, tolerated a regular diet by mouth, and she was safely discharged home.

**Figure 6:**
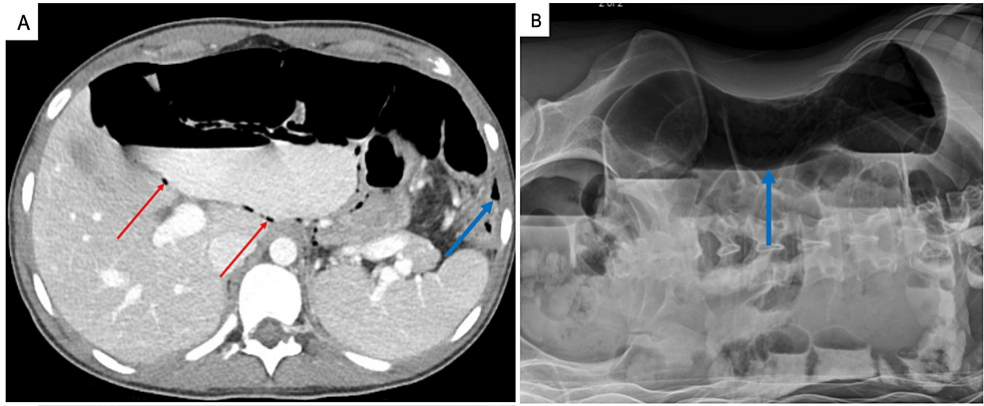
Computed tomography and abdominal radiograph demonstrating pneumoperitoneum and dilated loops of bowel A) Computed tomography axial image demonstrating multiple dilated loops of small bowel, intraperitoneal free air (blue arrow) near the left paracolic gutter concerning for perforated viscus, and pneumatosis intestinalis (red arrows) involving a small bowel loop.  B) Flat and upright abdominal radiograph demonstrating a large-volume pneumoperitoneum (blue arrow) and moderately dilated loops of the small bowel.

She felt well for one month and was seen in clinic, but then returned to our hospital with several days of intermittent abdominal pain and belching. She did not have any vomiting and was passing stool and flatus. Her CT scan and abdominal radiograph were concerning for significant small bowel pneumatosis, dilated small bowel, and pneumoperitoneum of unknown etiology (Figure 6). However, her physical exam revealed minimal abdominal distension with mild tenderness. Her white blood cell count, serum lactate levels, and liver function tests were normal. Despite her well-appearing condition, we decided to proceed with an exploratory laparotomy as there was no good explanation for her recent abdominal pain and abnormal CT and radiograph findings.

Upon exploration, we encountered dense adhesions and what appeared to be a partial volvulus of the small bowel. After a careful lysis of adhesions and inspection of the bowel, the entire bowel was viable, and no resection was performed. The pneumatosis was likely caused by intermittent volvulus resulting in a compromised blood flow. Postoperatively, she recovered well and was again discharged home.

Over one year has passed and she has continued to do well without recurrence of abdominal complaints. She has been under the care of a gastroenterologist and is gaining weight appropriately on a high-calorie nutrition regimen and vitamin D supplementation.

## Discussion

The etiology of megaduodenum is multifaceted as it is driven by either mechanical or functional abnormalities depending on the presence of a physical duodenal obstruction [1-4]. When there is no identifiable mechanical or functional abnormality, this presentation is referred to as idiopathic megaduodenum. Furthermore, megaduodenum can also be categorized as either congenital or acquired [1-4].

Mechanical megaduodenum is caused by an obstruction at the distal end of the duodenum [1-4]. It can be due to duodenal wall abnormalities (e.g., duodenal inflammation or diverticulum), duodenal lumen abnormalities (e.g., atresia, congenital stenosis, webs, gallstones, bezoars, fecal calculus, duodenal diaphragm, or parasites), external compression of the duodenum (e.g., annular pancreas, congenital bands, tumors, or SMA syndrome) [1-6]. Congenital malformations, such as congenital stenosis and bands, are the main cause of mechanical megaduodenum in childhood [4]. In SMA syndrome, the third portion of the duodenum is compressed by the overlying SMA leading to duodenal obstruction and dilation. 

In the absence of mechanical obstructive factors, functional or nonobstructive abnormalities can lead to functional megaduodenum, the true etiology of which remains unknown [1-4]. However, it is theorized that neuropathic and myopathic disorders that lead to mproper duodenal motility may lead to functional megaduodenum [4]. These include congenital diseases (e.g., congenital ganglion cell deficiency, ganglion cell dysplasia, duodenal aganglionosis, hereditary megaduodenum, porphyria, abnormal distribution of cell of Cajal, and collagen diseases) and acquired diseases (e.g., diabetes mellitus, systemic lupus erythematosus, amyloidosis, scleroderma, polymyositis vitamin deficiency, Chagas disease, and post-vagotomy intestinal paralysis), which affect the function of the intestinal wall plexus or the smooth muscles [1-4,7].

On the other hand, idiopathic megaduodenum is a diagnosis of exclusion. While the etiology of idiopathic megaduodenum is unknown, undiagnosed abnormal gut collagen and interstitium or neuromuscular disorders are suspected to cause decreased peristalsis and accumulation of motilin in the duodenal cavity [1]. Histopathologic analysis did not demonstrate any of these in our patient.

The clinical manifestation of megaduodenum can be nonspecific and lead to delays in diagnosis [4,5]. In all forms of megaduodenum, the foundational problem is a failure of food contents to transit through the duodenum [1-4]. Duodenal stasis leads to retention of incompletely digested duodenal contents, which causes dilation of the duodenum, repeated nausea and vomiting, and subsequent electrolyte abnormalities, dehydration, weight loss, and malnutrition [1,5]. The incompletely digested contents lead to increased oncotic pressure within the duodenum, resulting in chronic diarrhea that further worsens weight loss, malnutrition, and dehydration [1]. One of the clinical manifestations of megaduodenum associated malnutrition is pellagra, caused by vitamin B3 (niacin) deficiency [6]. Patients with megaduodenum may also experience abdominal distension, excessive salivation, hematemesis, recurrent acute appendicitis, steatorrhea, epigastric abdominal pain, dyspepsia, osteoporosis, gastric plenitude, and early satiety [4,7].

Given the nonspecific clinical manifestation of megaduodenum, its differential diagnosis is broad and includes Chaga’s disease, scleroderma, Hirschsprung’s disease, chronic idiopathic intestinal pseu­do-obstruction, intestinal amyloidosis, achalasia, congenital bands, duodenal atresia, foreign bodies, adhesions, tumors, and peptic ulcer [1-5]. Diagnostic modalities, such as abdominal radiographs, upper gastrointestinal contrast radiograph series, endoscopy, ultrasound, and computed tomography, may aid with diagnosis and surgical planning [1-5,7-9]. However, an official diagnosis and classification of megaduodenum is best accomplished through an exploratory laparotomy with biopsy and subsequent histopathologic analysis to identify all underlying factors [8,9].

The treatment of megaduodenum depends on the underlying cause and degree of duodenal distention. For megaduodenum secondary to other diseases, the primary disease must be addressed [2]. Medical management includes supportive therapy with frequent small feedings, maintenance of electrolyte imbalances, broad-spectrum antibiotics to limit bacterial overgrowth and dietary management with low-fiber, high-calorie diets, and nutritional supplementation with total parenteral nutrition [5,9]. While medical management helps to minimize patient symptoms and facilitates nutritional rehabilitation, medical management alone yields limited benefits and poor outcomes. Surgery is inevitable and is considered the treatment of choice for megaduodenum [1,5,9]. Many corrective surgical options have been reported in the literature and include tapering duodenoplasty plus roux-en-Y duodenojejunostomy, end-to-side gastrojejunostomy or duodenojejunostomy, duodenectomy with feeding jejunostomy, side-to-side gastrojejunostomy plus tapering duodenoplasty, feeding jejunostomy with draining gastrostomy, partial gastrectomy with duodenectomy, partial duodenal resection, and subtotal duodenectomy with jejunal patch [1-5,9].

No one procedure has been identified as superior to the rest. Regardless of which surgical option is chosen, the ultimate goals of surgery are as follows: first, to restore GI tract continuity and provide a reliable unobstructed path for duodenal drainage to prevent duodenal retention and stasis; second, to reduce the capacity of the enlarged atonic duodenum; and finally, to prevent forceful feeding of the duodenum [1,3]. To prevent reoccurrence of megaduodenum and its associated duodenal stasis retention and subsequent duodenal dilation, it is crucial that all three goals are accomplished by the chosen surgical option [1]. For our patient, duodenal plication is the preferred surgical procedure, which provides the least disruption to normal anatomy. It is important to ensure the restoration of intestinal continuity and ensure that functional biliary drainage is maintained [3].

## Conclusions

Megaduodenum is a rare clinical syndrome with nonspecific clinical manifestations, making it a challenging diagnosis. Delay in surgical care can lead to increased morbidity. Diagnosis and accurate classification require surgical exploration of the abdomen with a full-thickness biopsy and subsequent histopathologic analysis to rule out any obstructive or functional disorders of the duodenum as seen in mechanical megaduodenum and functional megaduodenum, respectively. The treatment of megaduodenum depends on the underlying cause and degree of duodenal distention. It is crucial that clinicians are knowledgeable of the various surgical options, their indications, and the potential postoperative complications.
